# Neuromuscular Junctions as Key Contributors and Therapeutic Targets in Spinal Muscular Atrophy

**DOI:** 10.3389/fnana.2016.00006

**Published:** 2016-02-03

**Authors:** Marina Boido, Alessandro Vercelli

**Affiliations:** Department of Neuroscience “Rita Levi Montalcini”, Neuroscience Institute Cavalieri Ottolenghi, University of TorinoTorino, Italy

**Keywords:** motor neuron disease, endplate, denervation, immaturity, neurofilament, therapy

## Abstract

Spinal muscular atrophy (SMA) is a recessive autosomal neuromuscular disease, representing the most common fatal pediatric pathology. Even though, classically and in a simplistic way, it is categorized as a motor neuron (MN) disease, there is an increasing general consensus that its pathogenesis is more complex than expected. In particular, neuromuscular junctions (NMJs) are affected by dramatic alterations, including immaturity, denervation and neurofilament accumulation, associated to impaired synaptic functions: these abnormalities may in turn have a detrimental effect on MN survival. Here, we provide a description of NMJ development/maintenance/maturation in physiological conditions and in SMA, focusing on pivotal molecules and on the time-course of pathological events. Moreover, since NMJs could represent an important target to be exploited for counteracting the pathology progression, we also describe several therapeutic strategies that, directly or indirectly, aim at NMJs.

## Physiological Development and Muscle Innervation at the Neuromuscular Junction

Neuromuscular junctions (NMJs) are specialized synapses in the peripheral nervous system, which allow the transmission between the motor nerve terminal and skeletal muscle fibers, consequently inducing muscle contraction. In this review we will mostly refer to rodent NMJs, since the majority of the available data in the literature usually come from experimental models, despite some differences with human NMJs (see below).

The mechanisms leading to muscular innervation involve a huge number of molecules, among which acetylcholine/acetylcholine receptors (ACh/AChRs), that are pivotal in the impulse transmission, and several other proteins, that play a crucial role in the development/maintenance/maturation of NMJs (as agrin, MuSK, rapsyn, etc.). These molecules will be described below.

NMJ development begins at the embryonic days (E) 12–14 in the mouse, until the stabilization of mature endplate at E17 (Witzemann, 2006). Then, during postnatal development, further changes in NMJ structure occur: the initial uniform receptor density in clusters is progressively rearranged and transformed in multi-perforated and invaginated structures, acquiring the characteristic “pretzel like” pattern (Slater, 1982; Marques et al., 2000).

According to the classical model of NMJ development, NMJ location depends on the random initial contact of the nerve terminal onto the developing muscle fibers, leading to the AChRs clustering (“agrin hypothesis”, McMahan, 1990). However this view has been recently revised: NMJ development may require the prepatterning of AChRs on muscle membrane where the outgrowing motor neuron (MN) fibers will synaptically contact the muscle. This means that AChR clusters are present on the muscle surface before the motor fiber arrival, and that their initial position probably influences the position of nerve–muscle contacts (Vock et al., 2008): this theory (“myocentric model”) contemplates a nerve-independent AChR prepatterning at the muscular level, depending on muscle-specific regulators (as MuSK, and rapsyn, see further; Witzemann, 2006; Tintignac et al., 2015). However, between these two alternative models, the actual mechanisms are probably in the middle (Tintignac et al., 2015).

Concerning the innervation, all muscle fibers undergo an initial step of poly-innervation, followed by a postnatal withdrawal of nerve terminals leading to motor neuronal mono-innervation (notably in humans the withdrawal of motor axon terminal occurs before birth; Macintosh et al., 2006). The underlying mechanisms of such process probably depend on the synaptic activity and on the competition between nerve terminals (involving both Hebbian potentiation of active synapses, and synaptotoxic/synaptotrophic cues in NMJ stability): indeed inactive NMJs undergo a reduction of post-synaptic specializations, whereas increasing activity induces monoinnervation. Moreover, when a motor terminal is stabilized, the neighboring inactive ones are eliminated: they receive “punishment signals” released by active MNs, that determine their elimination (Walsh and Lichtman, 2003; Bloch-Gallego, 2015). Synapse elimination covers a gradual process of withdrawal (presence of retraction bulb) and atrophy of the nerve terminal (Buffelli et al., 2004).

Several molecules contribute the development and stabilization of NMJs. One key molecule is agrin, a heparan sulfate proteoglycan synthesized and released by neurons, muscle fibers and Schwann cells (Ruegg et al., 1992): neural agrin has been described as 1000-fold more effective in stimulating AChR clustering than that synthesized by other cells (Reist et al., 1992). Depending on the theories mentioned above, agrin can be considered both an inducer of AChR clustering and a stabilizer of the post-synaptic membrane (Witzemann, 2006); it triggers the autophosphorylation of the muscle-specific tyrosine kinase receptor (MuSK), and they interact with each other via a transmembrane protein named low-density lipoprotein receptor related protein 4 (LRP4; Kim et al., 2008), determining the AChR clustering through the cytoplasmic linker protein rapsyn. Indeed the knockout mouse models for either agrin or MuSK similarly cause dramatic reduction and alterations of AChRs, leading to perinatal death (DeChiara et al., 1996; Gautam et al., 1996).

Other “auxiliary proteins” (including neuregulins, dystrophin-glycoprotein complex, ErbB receptors, and Wnts) participate in the development and maintenance of NMJs (for an extensive review, refer to Tintignac et al., 2015). Since the NMJ formation is partially regulated by different molecules in rodents and humans (for example, 19 different Wnts), this process is probably differently orchestrated among species (Wu et al., 2010). Finally microRNAs (miRNAs) could play an important role in NMJ formation, functioning and/or synaptic homeostasis as well, in both invertebrates and vertebrates (Kye and Gonçalves Ido, 2014). For example, Drosophila mutants that lack miR-125 showed a size reduction of NMJs and delayed endplate maturation, compared to aged-matched controls (Caygill and Johnston, 2008). In addition, in mammals, some miRNAs seem to be implied either in the correct synaptic function in the NMJs (miRNA-124; Zhu et al., 2013), or in their reinnervation after injury/during MN disease (miRNA-206; Williams et al., 2009).

Increasing evidence suggest that NMJs can represent an early pathological target in several neuromuscular diseases, including spinal muscular atrophy (SMA) and amyotrophic lateral sclerosis (ALS), although they are historically considered MN-centered pathologies. Here we intend to describe and analyze the vulnerability of SMA endplates, also discussing the potential therapeutic approaches that specifically target NMJs.

## Spinal Muscular Atrophy: A Brief Description of Pathogenic Mechanisms

SMA is a recessive autosomal neuromuscular disease and the most common fatal pediatric pathology, with an incidence of around 1 in 10,000 live births and a carrier frequency of 1:31 (Prior et al., 2010).

SMA is caused by the deletion/mutation of the survival motoneuron gene (*SMN*). In humans, there are two *SMN* genes, the telomeric *SMN1* coding for an ubiquitous protein (full-length SMN or FL-SMN), and its centromeric homolog *SMN2* mostly generating a protein lacking exon 7 (Δ7-SMN), which is not functional: indeed *SMN2* gene produces a limited amount of functional protein which can modulate SMA severity (Lorson et al., 1999). This accounts for the presence of four main clinical SMA types (I-IV), characterized by different age of onset and disease severity (Lefebvre et al., 1995; see Table 1). The genetic defects occurring in SMA determine the degeneration of spinal MNs, leading to a progressive muscular atrophy and in the most severe cases, to death.

**Table 1:** **Classification of human SMA forms (according to Lefebvre et al., 1995; Feldkötter et al., 2002; D’Amico et al., 2011)**.

	SMN2 copy number	Disease onset	Highest motor function achieved	Clinical features	Average survival
Type I (severe; Werdnig- Hoffmann disease)	1–2	0–6 months	Never sit unaided	Hypotonia; symmetrical flaccid paralysis; limited head control; poor spontaneous motility; tongue fasciculation; respiratory distress	<2 years
Type II (intermediate)	3	7–18 months	Sit; Never stand unaided	Tremors of upper extremities; deep tendon reflex lack; joint contractures; kyphoscoliosis; masticatory muscle weakness	10–40 years
Type III (mild; Kugelberg- Welander disease)	3–4	>18 months	Stand and walk (depending on the disease severity)	Muscular weakness (depending on the disease severity)	Adult
Type IV (adult)	>4	20–30 years	Walk unaided	No relevant motor symptoms; no respiratory and nutritional problems	Adult

As reviewed by Li et al. (2014), SMN is a 38 kD protein and accumulates into nuclear structures named Gems: here SMN can associate with eight other proteins (Gemins2–8 and Unrip) to form a large macromolecular complex. Its role concerns the biogenesis/assembly of small nuclear ribonucleoproteins (snRNPs), the pre-mRNA maturation and the axonogenesis (Fallini et al., 2012). Lack of SMN determines specific MN death, although the reasons for this selectivity remain unclear: on one hand, the disrupted formation snRNPs could affect the splicing of some genes involved in MN circuitry; on the other, SMN could play a role in axons (including support in axonal growth and branching, RNA metabolism and transport), lacking in SMA. Probably these two hypotheses are intertwined, that is, reduced snRNP assembly could influence the splicing of genes important for axons (Burghes and Beattie, 2009; Fallini et al., 2012). Moreover SMN has been also found localized in presynaptic terminals at the NMJ, and interacts directly with heterogeneous nuclear ribonuclear protein R, suggesting its crucial role in recruiting and transporting RNA particles into axons and axon terminals (Dombert et al., 2014).

Indeed increasing evidence suggest that SMA pathogenesis is more complex than expected: many authors have recently speculated that, even though MNs are the most affected cells in SMA, their loss might not only depend from the lack of SMN: retrograde signals coming from muscles and NMJs can be crucial players of the MN alteration (Bottai and Adami, 2013). Indeed several alterations have been described at peripheral level.

## NMJ Alterations in SMA

**Figure 1:**
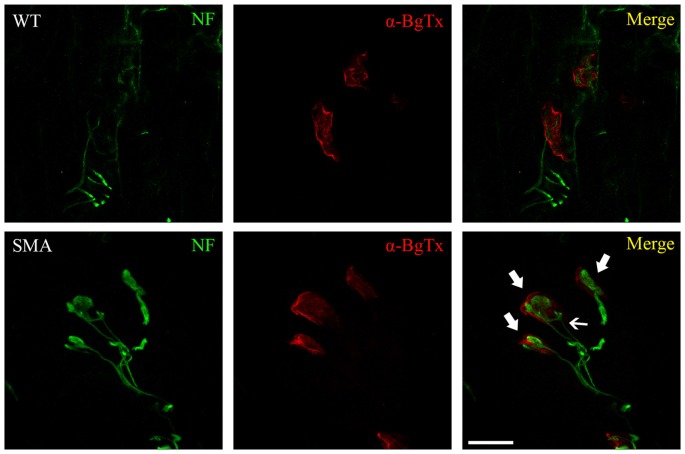
**WT and spinal muscular atrophy (SMA) neuromuscular junctions (NMJs) in P10 quadriceps of SMNΔ7 SMA mice.** Immunofluorescence against neurofilament (NF, green) and α-bungarotoxin (α-BgTx, red) reveals dramatic alterations at SMA NMJ level, compared to WT: endplates are immature (less perforated in comparison to control mice), are completely engulfed by NF (thick arrows) and sometimes receive multiple innervation (thin arrow; Valsecchi et al., 2015). Scale bar = 20 μm for WT, 15 μm for SMA.

Both human and murine SMA NMJs show three main pathological features, including immaturity, denervation and neurofilament (NF) accumulation (Figure 1). The morphology and the functionality of endplates can be easily evaluated by α-bungarotoxin (α-BgTx) staining, that binds AChRs of skeletal muscles, in association to an immunoreaction against NF, which can highlight the NMJ innervation.

In agreement with other authors (Cifuentes-Diaz et al., 2002; Kariya et al., 2008; Kong et al., 2009; Ruiz et al., 2010), we observed that SMNΔ7 SMA mice (one of the most used experimental models, resembling SMA type II) show dramatic abnormalities in the NMJ morphology: they are immature (perforation number is reduced), significantly smaller compared to WT, and sometimes fragmented (Valsecchi et al., 2015). Interestingly, this experimental model displays significant transcriptome changes, also regarding NMJ development: for example, the expression of Z+ agrin mRNA (required for NMJ maturation) is drastically reduced in SMA MNs (Zhang et al., 2013).

Therefore there is a defect in maturation of NMJs: in fact the fetal form of AChRs (gamma-AChRs) is still expressed at late stages of development (Kariya et al., 2008). Moreover at (postnatal day 14, P14) 50% NMJs are immature, compared to 10% of controls (Kariya et al., 2008). The results in the mouse models are confirmed in SMAI patients, in whom a decrease in NMJ area and number of perforations, and increased expression of the fetal gamma-AChR subunits have been described (Harding et al., 2015).

The lack of SMN seems to have a central role in NMJ formation and maturation. Interestingly, controlled knockdown of SMN in neonatal mice induced a severe neuromuscular disease phenotype, whereas the depletion of SMN after P17 in mice (when the fully NMJ maturation is established) had relatively low effect: moreover in adult SMN-depleted mice, a selective NMJ pathology occurred only in aged or injured animals. These results put again the NMJ at the core of SMA (Kariya et al., 2014). To further support the relationship between SMN and NMJ formation/maturation, NMJ-like structures were generated using MNs derived from SMA patient-specific induced pluripotent stem cells (iPSCs): the clustering of AChRs was strongly impaired (Yoshida et al., 2015).

Moreover such delayed maturation seems associated to an impaired synaptic function of motor terminals: indeed mutant mice show slowed postsynaptic potentials and reduced evoked neurotransmitter release (Ruiz et al., 2010). The reason of such compromised synaptic transmission could be related to a reduced rate of transport of synaptic vesicle 2-c and synaptotagmin, observed at P11 in SMNΔ7 SMA mice, before the reduction in synaptic vesicle density (Dale et al., 2011). Therefore the pathological findings in animal models suggest an impairment in NMJ function: repetitive nerve stimulation in SMA type II and III patients resulted in pathological decremental responses, suggesting that the functional abnormalities could be, at least in part, postsynaptic (Wadman et al., 2012).

These synaptic abnormalities appear early, before the motor symptoms onset, in SMNΔ7 SMA mice (Murray et al., 2008), and even at prenatal stages in Smn^−/−^;SMN2 mice, in which intercostal muscles show a very early denervation (E18.5)—suggesting that axon outgrowth and NMJ formation did not occur (McGovern et al., 2008). The symptoms become evident when about 50% endplates result denervated (Ling et al., 2012).

SMA is not the only neuromuscular disease with synaptic alterations: a comparison between ALS and SMA showed that, even though the involvement of muscle groups was similar in the two pathologies, in ALS the presynaptic alterations precede the postsynaptic ones, whereas in SMA they were coincident (Comley et al., 2015).

Another feature demonstrating an incorrect NMJ development concerns the persistence of polyneuronal innervation: such condition is generally associated to early stages of NMJ structuring, but is strongly delayed in SMAΔ7 mice (Lee et al., 2011).

These synaptic alterations could be related to the denervation affecting SMA muscles. Indeed Ling et al. (2012) performed an extensive analysis of about 20 muscles in the SMNΔ7 SMA mouse model and found a dramatic denervation in axial and appendicular muscles (in particular affecting muscles located in the head and trunk, and involved in maintaining head posture, respiration, mastication) at the late phase of the disease. This confirmed the hypothesis of Murray et al. (2008) suggesting that “muscle fiber-type and body location are likely to be important determining factors in regulating synaptic vulnerability during SMA”. Similarly, we observed that MNs innervating axial and proximal muscles are the most affected in SMNΔ7 SMA mice (d’Errico et al., 2013), confirming a selective weakness of these compartments. Other groups refer poor denervation in this mouse model, limited to intercostal and paraspinal muscles (Kong et al., 2009).

Notably it was demonstrated that muscles, even though initially innervated, lost the innervation during SMA progression, suggesting an impairment in synapse maintenance (Ling et al., 2012). The synaptic integrity of NMJs seems to depend upon levels of SMN produced by MNs, rather than by muscles (Martinez et al., 2012). Another possibility could be correlated with the impaired cellular transport in MNs: NF accumulation, resulting from dysregulation of the axonal transport machinery (Kreutzer et al., 2012), may have a role in the destabilization of NMJs. Interestingly it has been hypothesized that the reduction in SMN leads to disruption of β-actin mRNA transport and affects shuttling of NF within the axon of the MN, leading to aberrant NF accumulation and poor terminal arborization (Bowerman et al., 2007, 2009). Also the protein levels of dynein seem to be reduced, suggesting in turn an impairment in axonal transport (Dale et al., 2011).

Indeed NF accumulation is a pathological hallmark of SMA, since it has been found in the majority of affected muscles: its accumulation has been associated to the slow and delayed transport of components essential for NMJ maturation and maintenance (Torres-Benito et al., 2012). SMA NMJs are completely engulfed by NF, including its phosphorylated forms (Cifuentes-Diaz et al., 2002; Ling et al., 2012; Valsecchi et al., 2015), and this increases over time (Kariya et al., 2008); moreover this accumulation seems to be restricted to the endplate, sparing MN cell body, motor root and nerve (Cifuentes-Diaz et al., 2002; Dale et al., 2011), suggesting local alterations in NF dynamics. Nevertheless, retinal neuron primary culture, obtained from SMN-deficient mice, were characterized by NF aggregation into their neurites: the authors ascribed such alterations to transport defects, rather than to abnormalities in NF production (Liu et al., 2011).

Finally, Ling et al. (2012) also found that NF accumulates in both affected and spared muscles in SMNΔ7 SMA mouse model, and appears before the onset of denervation: therefore NF accumulation and denervation do not seem strictly correlated. However, NMJs of proximal muscles are affected by NF accumulation earlier than distal ones. Therefore such process could involve various muscles with a different timing, resulting for some reason more harmful for the proximal than the distal ones (Kariya et al., 2008). Could the extent of NF engulfment explain muscle impairment or sparing in SMA? The specific causes of such selectivity still remain unclear.

**Figure 2:**
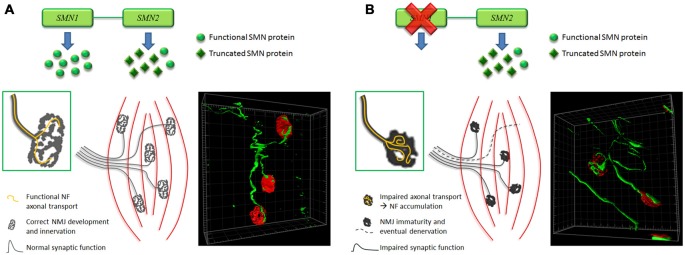
**Hypothesis of time-course of NMJ abnormalities affecting SMA muscles. (A)** In normal condition, a correct amount of SMN results in the development of mature NMJs, characterized by pretzel-like morphology and functional synaptic activity. Moreover isosurface reconstruction obtained by Imaris software (Bitplane) shows thin NF-positive fibers (green) innervating the NMJs (red). **(B)** Instead, in SMA, lack of SMN impairs axonal transport: this could determine NF accumulation at the NMJ level (as also shown by Imaris isosurface rendering), affecting their correct maturation and innervation (endplates are smaller, less perforated and sometimes denervated), and consequently their synaptic function. Such alterations could backwards damage motor neurons (MNs; Valsecchi et al., 2015).

In conclusion, even though the time-course of abnormalities affecting endplates remains matter of debate (Figure 2), there is a general consensus that the NMJ breakdown is an early event in SMA pathogenesis, which probably anticipates MN dysfunction and motor symptom onset. To this extent, NMJs could represent an important therapeutic target, to be exploited to counteract the pathology progression.

## Therapeutic Approaches

Even though for SMA no effective treatment is currently available, the efforts of researchers are constantly oriented to discover and test new drugs or compounds, at least aiming to delay the disease progression. A therapeutic strategy aimed at improving neuromuscular transmission (for example by increasing the release and the half-life of Ach) could be an effective and valid approach (Wadman et al., 2012).

Here we will focus on the therapeutic effects on NMJs, either when endplates represent the direct target of the treatment, or in case of systemic/less focused treatment also having an impact on NMJs. Additionally we will also mention still unexplored molecular targets in SMA.

### SMN Overexpression

Due to the central role of SMN in the SMA pathogenesis, many groups have evaluated the effects of its overexpression in mouse models. Moreover, some authors displayed that the synaptic connectivity at the NMJ level strictly depends on the presence of SMN in MNs, rather than in muscles: overexpression of SMN in MNs re-established the presynaptic properties of quantal content and the probability of synaptic vesicle release, with the consequent increase of the endplate size, the NF reduction and the denervation decrease (tibialis anterior and splenius capitis muscles; Martinez et al., 2012). Moreover the beneficial effects on rescuing endplate size and mitigating NMJ denervation status was obtained by restoring low levels of SMN (Paez-Colasante et al., 2013). However, selective overexpression of SMN in SMA neurons significantly reduced the NMJ defects (gastrocnemius, triceps and tibialis anterior muscles), but moderately improved motor performance and extended the lifespan of treated mice: despite the partially positive results, an ubiquitous restoration of SMN could have been more effective (Lee et al., 2012; Paez-Colasante et al., 2013). Moreover there is a time window in which SMN replacement is most beneficial to NMJs: indeed early P2 intracerebroventricular administration of scAAV9-SMN in SMNΔ7 SMA mice completely preserved the integrity and innervation of NMJs (longissimus capitis), compared to late (P7) administration (Robbins et al., 2014). Therefore even high SMN levels are ineffective in rescuing the phenotype, once the disease has reached advanced stages.

### Antisense Oligonucleotides (ASOs)

The use of ASOs represents another valid strategy to increase the levels of SMN, specifically restoring the SMN2 splicing pattern, activating the SMN2 promoter, and extending the half-life of SMN mRNA or protein (Tsai, 2012). Valproic acid (VPA) is currently one of the most studied ASOs: VPA administration in SMA type III mice improved motor performance, reduced NMJ denervation and muscular atrophy (Tsai et al., 2008). Moreover, *in vitro*, co-culturing C2C12 cells (myoblast cell line) and SMA patient-specific iPSCs resulted in a defective AChR clustering, that was improved by VPA administration, in association with the increase of SMNΔ7 and SMN-FL mRNA levels, but without NF decrease (Yoshida et al., 2015).

In a mouse model of the intermediate forms of SMA (Burgheron mutant) showing a delay in NMJ maturation and a decrease in the number of functional neuromuscular units, the systemic administration of SMN-restoring ASOs at the age of onset could extend survival and rescue the neurological phenotypes (Bogdanik et al., 2015).

Similar positive results has been also induced by trichostatin A injection, which increased innervation in severely affected muscles (longissimus capitis, splenius capitis, serratus posterior inferior and semispinalis capitis) in SMNΔ7 SMA mice: indeed denervation was reduced of about 90% in the most vulnerable muscles (Ling et al., 2012). Other ASOs (2′-O-2-methoxyethyl–modified ASO, Passini et al., 2011; 8-mer ASO, Keil et al., 2014) induced significant improvements in the NMJ morphology and innervation, in muscle physiology, motor performance, and survival respectively in SMNΔ7 SMA mice (quadriceps and intercostal muscles) and in the milder 5058-Hemi hybrid SMA mice (intercostal muscles).

Despite the impressive results offered by ASOs, translation to human patients still needs additional steps, in order to better establish timing, volume, and location of dosing, and to avoid toxic side effects (Porensky and Burghes, 2013).

### Neurotrophic Factors and Stem Cells

Impairment of AChR clustering in SMA is due to a delay in the switch from fetal gamma-subunit to the adult epsilon-subunit, normally occurring by 2 weeks after birth (Missias et al., 1996), and depending on neurotrophic factor release (Kues et al., 1995). Among neurotrophins, brain-derived neurotrophic factor (BDNF) and neurotrophin-3 (NT-3) can especially support functional maturation of NMJs (Wang et al., 1995). For this reason their replacement could be a valid therapeutic approach. Indeed in several experimental models (MN diseases as well as trauma), neurotrophic factors can exert general positive effects, including reduction of MN death, stimulation of axon regrowth, preservation of peripheral functional connections, NMJ maturation (Wyatt and Keirstead, 2010). In 1997, adenovirus-mediated gene transfer of NT-3 induced substantial therapeutic effects in a murine model of progressive motor neuronopathy, increasing animal survival and improving NMJ function (Haase et al., 1997). More recently, stem cell-derived MNs, transplanted in a murine model of SMA with respiratory distress type 1, appeared well integrated into the host, with axons reaching ventral roots and muscles, and forming new functional NMJs: this positive outcome can be ascribed to the neuroprotective effects exerted by graft, which even modulated inflammation (Corti et al., 2009). Also mesenchymal stem cells (MSCs) could be good candidates for MN disease cell therapy, since they display a great plasticity, secrete several growth factors [such as vascular endothelial growth factor, glial cell-derived neurotrophic factor (GDNF) and BDNF], modulate host immune system (Mazzini et al., 2009), and delay atrophy in denervated muscles (Jiang et al., 2012): allogenic MSCs have been administered to SMA type I patients, inducing improvements of general physical condition during treatment, without side effects (Villanova and Bach, 2015), but reference to NMJ feature was not reported.

On the other hand, the administration of a recombinant AAV vector encoding human insulin-like growth factor 1 (IGF-1) into the deep cerebellar nucleus of a SMA type III mouse model significantly reduced MN death, although without a correlated correction of muscle pathology or improvement of motor functions: in fact, analysis of the gastrocnemius NMJs revealed that the treatment did not rescue the denervation, since probably the survived MNs were not really functional and able to efficiently innervate the muscle (Tsai et al., 2012). However in our experience (Valsecchi et al., 2015), and in agreement with the literature (D’Amico et al., 2011), gastrocnemius, as a distal muscle, is generally less affected by SMA. Nevertheless the authors suggested to support such approach with SMN therapy, reinforcing the idea that SMN can positively influence the NMJ functionality (Tsai et al., 2012).

### miRNAs

Other promising therapeutic targets are represented by miRNAs, whose involvement in MN diseases is gradually emerging. Notably, SMN complex is involved in miRNA biogenesis and/or function, and increasing evidence demonstrated that miRNA dysregulation (regarding cell cycle, development, metabolism, and synaptic plasticity) affects SMA neurons and muscles (Kye and Gonçalves Ido, 2014; Valsecchi et al., 2015), even at embryonic age in SMNΔ7 SMA mice (Luchetti et al., 2015). In particular some miRNAs (miRNA-310, miRNA-125, let-7, miRNA-124) seem important players for both NMJ formation/function and synaptic homeostasis, in invertebrate and mammalian models (Kye and Gonçalves Ido, 2014). However, due to the relative novelty of such discoveries, few therapeutic approaches have been currently evaluated in SMA.

For example, miRNA-9 is down-regulated in MNs derived from SMN1^mut^ mESCs (Haramati et al., 2010) and in skin fibroblast cells of SMA patients (Wang et al., 2014): miRNA-9 is located downstream of SMN1 and can regulate the NF expression, suggesting its involvement in NF defects reported in SMA (Haramati et al., 2010). Additionally its expression seems to be related to SMA severity, making it a promising tool for SMA prognosis (Wang et al., 2014). An experimental modulation of the expression of miRNA-9 (gain- and loss-of-function studies) is still lacking, but should help in clarifying its role in SMA NF.

Also, deletion of miRNA-206 may accelerate ALS progression (G93A-SOD1 mice; Williams et al., 2009) and worsen dystrophic condition in an experimental model of Duchenne muscular dystrophy (Liu et al., 2012); miRNA-206 is also necessary to induce NMJ regeneration after sciatic nerve crush (Williams et al., 2009). It can effectively repress histone deacetylase 4 (HDAC4, known to inhibit muscle reinnervation; Bruneteau et al., 2013), resulting in the activation of FGF binding protein 1 (FGFBP1) which in turn boosts the FGF action during reinnervation (Williams et al., 2009). We have recently demonstrated that the same pathway is activated in SMNΔ7 SMA mice (Valsecchi et al., 2015): as in ALS, miRNA-206 may play a compensatory role in reinnervation, acting as a survival endogenous mechanism, although not sufficient to prevent the disease progression (Williams et al., 2009; Liu et al., 2012; Valsecchi et al., 2015). Finally it is worth noting that, although other miRNAs could contribute to formation of NMJ and muscle reinnervation (miRNA-133b and miRNA-1, that together with miRNA-belong to the so called “myomiR” network; van Rooij et al., 2008), the only miRNA-206 seems essential in maintaining and repairing endplates (Valdez et al., 2014). Indeed the delivery of miRNA-206 mimics or the modulation of its downstream targets could be beneficial for SMA and similar neuromuscular diseases.

### Agrin

Agrin is one of the fundamental molecules in stabilizing synaptic contacts and controlling axon growth (Witzemann, 2006). Interestingly SMNΔ7 SMA MNs lack neuronal (Z+) agrin protein at P1, and are completely deficient at P3 (Zhang et al., 2013). Moreover cultured skeletal muscle cells isolated from SMA type I patients show abnormal fusion mechanisms and impaired agrin-induced AchR aggregation, proving that NMJs cannot be correctly built (Arnold et al., 2004). Therefore agrin could be an interesting therapeutic target. Agrin fragment administration determined NMJ stabilization and phenotype amelioration in SARCO mice (experimental model of Sarcopenia, characterized by age-related muscle wasting), and accelerated reinnervation after sciatic nerve crush, by activating the agrin/Lrp4/MuSK system (Hettwer et al., 2014). Moreover recently it has been demonstrated, in co-coltures of myotubes and PC12 cells, that muscular innervation (functional NMJ formation) was triggered when cells were treated with a combination of agrin and laminin, rather than only agrin, probably due to different signaling pathways underlying AChR clustering (Zhang et al., 2015). Additional *in vivo* studies may help in clarifying which therapeutic approach could be more efficient for SMA or similar neuromuscular diseases, in order to support functional neuromuscular development/restoration.

### Exercise

Even though historically physical activity was not recommended to neuromuscular disorder patients (including SMA) dreading the risk of further muscle damage (Bennett and Knowlton, 1958), nowadays strong evidence reversed this opinion. Exercise can exert beneficial effects on muscle and NMJ morphology/function, during aging and in case of peripheral nerve injury or degenerative pathologies. As reviewed by Nishimune et al. (2014), exercise can induce the activation of several molecules, including extracellular matrix molecules (laminin), neuregulins (Neuregulin-1, PGC-1a) and neurotrophic factors (BDNF, GDNF, IGF), and modulate miRNA expression (miRNA-206), positively influencing NMJ morphology and transmission, their maintenance and regeneration. Indeed, neuromuscular system is strongly influenced by exercise, able to induce both structural and functional modifications; physical activity can also favorably support AChR assembly (Ferraiuolo et al., 2009). Therefore, using exercise as a trigger of these molecular pathways could represent a valid therapeutic approach for MN/neuromuscular diseases or age-related atrophic pathologies. For example, old mice undergone wheel running training (in association with diet caloric restriction) displayed a reduced number of fragmented and denervated NMJs in the tibialis anterior, gracilis and gastrocnemius muscles (Valdez et al., 2010). Moreover, trained (running) type II SMA-like mice exhibited improved motor performance, diminished muscle atrophy and reduced MN death, compared to sedentary mice (Grondard et al., 2005). Better understanding the molecular pathways activated by exercise at the muscular and NMJ level in SMA could be extremely useful. Indeed physical activity should be associated to other therapies, to obtain a synergistic effect.

## Conclusion

The role of NMJs in the pathogenesis of SMA is anything but peripheral, so that pathological changes at the endplate level even precede the MN loss.

However divergent data are often present in the literature, and a more extensive analysis is therefore needed to better clarify the molecular, functional and temporal defects affecting NMJs, and consequently muscles and MNs.

As shown, numerous therapeutic approaches (stem cells, miRNAs, ASOs, etc.) have been tested either directly in SMA or in other similar neuromuscular/atrophic diseases. The obtained results are generally promising, but additional studies are necessary in view of a translational approach.

Indeed many crucial points still need to be unravelled, concerning the overall SMN function, the selective vulnerability of proximal muscles and MNs, the precise time-course of pathological events, the correlation among NMJ, muscle and neuron defects. Only the answers to these critical questions will allow to identify the best and most efficient therapy/ies for SMA.

## Author Contributions

MB has conceived and written the review. AV has revised and discussed the article.

## Funding

This work was supported by funds from RF-2009-1475235 (Italian Ministry of Health), Girotondo/ONLUS and Smarathon-ONLUS associations to AV, and by University of Turin (ex 60% Linea B 2013) grant to MB.

## Conflict of Interest Statement

The authors declare that the research was conducted in the absence of any commercial or financial relationships that could be construed as a potential conflict of interest.
